# Immune checkpoint inhibitors alone vs immune checkpoint inhibitors—combined chemotherapy for NSCLC patients with high PD-L1 expression: a network meta-analysis

**DOI:** 10.1038/s41416-022-01832-4

**Published:** 2022-05-31

**Authors:** Yimin Wang, Hedong Han, Fang Zhang, Tangfeng Lv, Ping Zhan, Mingxiang Ye, Yong Song, Hongbing Liu

**Affiliations:** 1 Department of Respiratory Medicine, Jinling Hospital, Nanjing Medical University, Nanjing, China; 2 Department of Respiratory Medicine, Jinling Hospital, Nanjing University School of Medicine, Nanjing, China

**Keywords:** Non-small-cell lung cancer, Cancer immunotherapy

## Abstract

**Background:**

We indirectly compared the effects of immune checkpoint inhibitors alone (ICI) and ICI-combined chemotherapy (chemo-ICI) in patients with non-small cell lung cancer who had high programmed death-ligand 1 (PD-L1) expression (defined as tumour proportion score ≥50% or TC3/IC3) through network meta-analyses.

**Methods:**

Through literature searches, we shortlisted 22 randomised controlled trials encompassing 4289 patients, with objective response rate (ORR), progression-free survival (PFS), and overall survival (OS) set as the primary outcomes. The dichotomous data for ORR and hazard ratios (HRs) and their 95% confidence intervals (CIs) for OS and PFS were extracted.

**Results:**

We found that chemo-ICI had significantly improved ORR (OR 1.7, 95% CI 1.1–2.5) and PFS (HR 0.59, 95% CI: 0.48–0.74) relative to ICI. Although no significant difference in OS was observed, the analyses revealed that the chemo-ICI patients tended to undergo fewer progression events than ICI patients (HR 0.82, 95% CI 0.6–1.1). In subgroup analysis, the non-squamous, PD-1 inhibitor and first-line treatment cohorts exhibited significant differences in ORR and PFS, but not in OS. However, in the squamous, PD-L1 inhibitor, and previously treated cohorts, PFS, OS and ORR were not different between chemo-ICI and ICI patients.

**Conclusions:**

In conclusion, for non-squamous NSCLC patients, accepting PD-1 as the first-line treatment may be a relatively better option.

## Introduction

Lung cancer is the major cause of cancer-related deaths worldwide [1]. Non-small cell lung cancer (NSCLC) accounts for 85% of primary lung cancer, while squamous carcinoma accounts for nearly two-thirds of all NSCLC cases [2]. With the advancement in the research on NSCLC, a variety of treatment methods for corresponding mechanisms are being developed, which has contributed to gradually refining the treatment of NSCLC. For patients with NSCLC who had EGFR or ALK mutations, targeted therapy or chemotherapy is mostly used as the first-line therapy [3]. However, for those without EGFR or ALK mutations, chemotherapy remains the first-line therapy. With the wide adoption of immunotherapy, several new options have become available as the first-line treatment for patients with NSCLC, including the research on immune checkpoint inhibitors (ICIs) as the most matured and commonly used option [4].

PD-1 and PD-L1 inhibitors are the most widely used ICIs in the treatment of NSCLC. Particularly, several clinical studies have reported the efficacy of ICIs or ICI-combined chemotherapy (chemo-ICI) for the treatment of patients with NSCLC who had high programmed death-ligand 1 (PD-L1) expression [5–15]. These clinical trials define high PD-L1 expression as a tumour proportion score (TPS) of >50% or TC3/IC3. For example, KEYNOTE 024 indicated that the use of ICI alone had better efficacy than chemotherapy, in patients with NSCLC who had high programmed death-ligand 1 (PD-L1) expression [16]. In addition, subgroup analysis of the high PD-L1 expression NSCLC cohort in KEYNOTE 407 confirmed the effectiveness of immune checkpoint inhibitors combined with chemotherapy (chemo-ICI) [5]. Recently, RATIONALE 307 [17] and ORIENT-11 [18] established the clinical efficacy of Tislelizumab combined chemotherapy and Sintilimab combined chemotherapy, respectively. Empower Lung-1 proved the efficacy of Cemiplimab monotherapy [14].

However, head-to-head randomised controlled trials (RCTs) comparing ICI and chemo-ICI are lacking. Therefore, we adopted the NMA-based approach to compare the effect of ICI versus chemo-ICI in patients with NSCLC who had high PD-L1 expression [9]. Meanwhile, we also conducted some subgroup analyses to explore whether other factors contributed to the efficacy. Subgroup analysis was conducted according to the treatment lines across the entire cohort. Subgroup analysis by histological type was also performed. Furthermore, PD-1 and PD-L1 inhibitors were categorised for subgroup analysis.

## Methods

### Search strategies

We searched all RCTs related to NSCLC from PubMed, Cochrane Library, Embase, and other databases from inception until March 2021, with no start data limit applied. In addition, we also performed a manual search for the reference lists across all available original studies, reviews, and meeting reports from the main international lung cancer meetings. The keywords used to search included: “non-small cell lung cancer”, “non-squamous lung cancer”, “nivolumab”, “pembrolizumab”, “atezolizumab”, “PD-1 inhibitor”, “PD-L1 inhibitor”, “programmed cell death-Ligand 1”, “cemiplimab” and others (Supplemental Methods). The language the RCTs used was limited to English. Two authors accomplished the search independently, and any discrepancy was figured out by mutual discussion to reach a consensus.

### Selection criteria

According to the PICOS framework, papers that conformed to the following criteria were included: (I) only NSCLC patients, representative of high PD-L1 expression cohort. (II) Studies that included the ICI cohort and chemotherapy cohort or included the Chemo-IC cohort and the chemotherapy cohort. (III) Studies that included a comparison of the ICI cohort with the chemotherapy cohort or chemo-ICI cohort with the chemotherapy cohort. (IV) Studies that reported outcomes including more than one of the following: ORR, PFS, OS and hazard ratios (HRs) and their 95% confidence intervals (CIs) for OS and PFS. (V) Studies that were all RCTs.

The following were the exclusion criteria: (I) Patients who had previously undergone systemic immunosuppressive therapy or active autoimmune disease. (II) Studies that included trials with radiotherapy as an intervention. (II) Case–control studies, retrospective studies, cohort studies, case reports, meta-analyses and systematic reviews were excluded.

### Data extraction

Two authors independently examined the title, summary, full text and supplementary materials to evaluate the eligibility and collect data from the included papers. The data were extracted into a spreadsheet. The data extracted for critical appraisal included the country clinical trial number, year of publication, first author, intervention, study stage, the number of patients in each group, and participant characteristics (if any). In addition, data on OS, PFS and ORR were extracted on a spreadsheet; it also included HRs and their 95% CIs for OS and PFS. Similarly, dichotomous ORR data were clustered. We treated the items as NR (not reported) if any of the categories above were not reported data in the RCTs.

### Quality assessment

The Cochrane Collaboration’s Risk of Bias tool was used to evaluate the methodological quality of the included RCTs. Factors such as randomness, double-blindness, the integrity of the outcome data, and bias in selective reporting were evaluated. The risk of bias was assessed according to the following criteria: low risk, high risk and ambiguous risk. The two authors completed this work independently and resolved all disputes by discussion.

### Statistical analysis

The HRs for PFS and OS as well as odds ratios (ORs) for ORR were primary endpoints in this network meta-analyses. A Bayesian approach was accordingly adopted. The survival analysis of PFS and OS were presented as HRs and their 95% CIs. ORR were treated as dichotomous variables; therefore, odds ratios (ORs) were used to present these parameters. We used the *χ*^2^ test and *I*^2^ statistics to evaluate the statistical heterogeneity of the included studies. A fixed-effects model would be selected to count the pooled estimate, if the *P* value for *χ*^2^ > 0.1 and *I*^2^ was <50% [19]. If not, a random-effects model would be used to combine the studies. When I^2^ statistic >50% or *P* value for *χ*^2^ < 0.1, it would be considered to be statistically significant for heterogeneity. It would be considered no statistically significant difference when the 95% CI for indirect comparison comprised 1. Chemotherapy (arm C) was used as the common therapeutic arm in this adjusted indirect comparison. This network meta-analysis indirectly evaluated the relative efficacy of ICI versus chemo-ICI, via comparing ICI (arm A) with chemotherapy (arm C) and chemo-ICI (arm B) with chemotherapy (arm C) [20]. The following formula was used to estimate the result of log HR: log HRAB = log HRAC − log HRBC. The log HR of the regulated indirect comparison between arm A and arm B was presented as log HRAB. Similarly, the log HR of the regulated indirect comparison between arm A and arm C was presented as log HRAC, while the log HR of regulated indirect comparison between arm B and arm C was presented as log HRBC. The formula: SE (log HRAB) = √SE(log HRAC)^2^ + SE(log HRBC)^2^ was used to estimate standard error (SE) [20]. ORs and the corresponding SEs were similarly calculated. The Bayesian network meta-analysis estimated the relative treatment effects by HRs and their 95% CI values. The treatment effects ranking of all comparison variables (PFS, OS and ORR) indicated the possibilities. For each upshot, three Markov chains with dissimilar starting values were run in parallel for 100,000 iterations, and each chain used a thinning interval of 10 and 10,000 burn-ins. The NMA computed the possibilities of each therapy being the best among all therapies by ranking the effects of all therapies in each iteration and then calculating the percentage of each therapy being ranked first across all iterations (where, 0 = a therapy that is certain to be the worst, 1 = a therapy that is certain to be the best). Subgroup analyses were conducted according to the treatment lines across the entire cohort. Non-squamous or squamous NSCLC and PD-1 inhibitor or PD-L1 inhibitor were also categorised for subgroup analyses. Based on the pre-arranged grouping factors, we collected the data of relevant subgroups in all included trials. We used the funnel plot and Eegg and Bgger tests to evaluate publication bias. All statistical analyses were executed using the R (version 4.0.5) and R Studio software. Gemtc package contributed to the statistical analyses.

## Results

### Studies included in the meta-analysis

We searched 11,459 associated publications in the beginning, and all of these were from ClinicalTrials.gov and contained conference abstracts. Subsequently, 3272 publications were removed because of duplicates, and 8187 articles were screened. After reading the titles, 8024 articles were excluded because of unrelated topics. Upon scrutinising the abstracts of the remaining 163 articles, we eliminated 34 systematic reviews, 68 retrospective studies, and 29 meta-analyses. After examining the full text, 32 articles were finally evaluated for eligibility. Among these, six trials lacked the high PD-L1 expression group and another six trials had unexpected interventions, including ipilimumab and bevacizumab. In the end, 22 RCTs were included in this NMA. The selection flowchart for the searched articles is presented in Supplemental Fig. 1. Among the 22 RCTs, 11 were related to ICI alone versus chemotherapy (*n* = 3113 patients) and 11 were on chemo-ICI (*n* = 1176 patients). Of the 11 RCTs that involved ICI intervention, 4 were on atezolizumab, 3 were on pembrolizumab, 1 was on avelumab, 1 was on cemiplimab, 1 was on durvalumab, and 1 was on nivolumab. In the 11 RCTs that had a chemo-ICI intervention, 3 were on chemo-pembrolizumab, 2 were on chemo-atezolizumab, 2 were on chemo-tislelizumab, 2 were on chemo-sintilimab, and 2 were on chemo-camrelizumab. The risk assessed by the risk of bias evaluation was within acceptable limits. The primary baseline characteristics of the 14 applicable studies are described in Table 1.

**Table 1:** Primary characteristics and the results of the applicable studies.

Study	Year	Fiest-authour	Treatment	PD-L1/PD-1	Number of events/Number of patients	ORR	PFS HR 95% CI	OS HR 95% CI
keynote-010-1 [26]	2016	Roy S Herbst	ICI	PD-1	42/139	30.2	0.59 (0.44–0.78)	0.54 (0.38–0.77)
			chemotherapy		12/152	7.9		
keynote-010-2 [26]	2016	Roy S Herbst	ICI	PD-1	44/151	29.1	0.59 (0.45–0.78)	0.50 (0.36–0.70)
			chemotherapy		12/152	7.9		
keynote-021	2016	Corey J Langer	ICI + chemo	PD-1	16/20	80	NR	NR
			chemotherapy		6/17	35		
keynote-024	2016	Martin Reck	ICI	PD-1	69/154	44.8	0.50 (0.37–0.68)	0.60 (0.41–0.89)
			chemotherapy		42/151	27.8		
keynote-042	2019	Tony SK Mok	ICI	PD-1	118/229	39	0.81 (0.67–0.99)	0.69 (0.56–0.85)
			chemotherapy		96/300	32		
keynote-407	2020	Luis Paz-Ares	ICI + chemo	PD-1	44/73	60.3	0.37 (0.24–0.58)	0.64 (0.37–1.10)
			chemotherapy		24/73	32.9		
IMpower110	2020	Roy S Herbst	ICI	PD-L1	41/107	38.3	0.63 (0.45–0.88)	0.59 (0.40–0.89)
			chemotherapy		28/98	28.6		
IMpower130	2019	Howard West	ICI	PD-L1	NR/88	NR	0.51 (0.34–0.77)	0.84 (0.51–1.39)
			chemotherapy		NR/42	NR		
IMpower 131	2020	Robert Jotte	ICI + chemo	PD-L1	29/47	61.7	0.44 (0.27–0.71)	0.48 (0.29–0.81)
			chemotherapy		14/44	31.8		
IMpower 132	2020	Makoto Nishio	ICI + chemo	PD-L1	18/25	72	0.46 (0.22–0.96)	0.73 (0.31–1.73)
			chemotherapy		11/20	55		
IMpower189 [27]	2020	L Gandhi	ICI + chemo	PD-1	81/132	61.4	0.36 (0.26–0.51)	0.42 (0.26–0.68)
			chemotherapy		16/70	22.9		
CheckMate 026 [28]	2017	DP Carbone	ICI	PD-1	35/88	34	1.07 (0.77–1.49)	0.90 (0.63–1.29)
			chemotherapy		49/126	39		
Camel	2020	Caicun Zhou	ICI + chemo	PD-1	NR/30	NR	0.39 (0.14–0.99)	NR
			chemotherapy		NR/20	NR		
Camel-sq	2021	Caicun Zhou	ICI + chemo	PD-1	NR/37	NR	0.30 (0.17–0.55)	0.48 (0.21–1.12)
			chemotherapy		NR/44	NR		
Empower-lung 1	2021	Ahmet Sezer	ICI	PD-1	111/283	39	0.54 (0.43–0.68)	0.57 (0.42–0.77)
			chemotherapy		57/280	20		
JAVELIN Lung 200	2018	Fabrice Barlesi	ICI	PD-L1	42/168	25	0.65 (0.49–0.88)	0.67 (0.51–0.89)
			chemotherapy		15/147	10		
MYSTIC [15]	2020	Naiyer A Rizvi	ICI	PD-L1	NR/118	NR	0.76 (0.55–1.04)	0.76 (0.55–1.04)
			chemotherapy		NR/107	NR		
OAK	2016	Achim Rittmeyer	ICI	PD-L1	22/72	30.6	0.63 (0.43–0.91)	0.41 (0.27–0.64)
			chemotherapy		7/65	10.8		
ORIENT-11	2020	Yunpeng Yang, MD	ICI + chemo	PD-1	73/107	68.2	0.31 (0.20–0.49)	NR
			chemotherapy		24/61	39.3		
ORIENT 12 [29]	2021	Caicun Zhou, MD, PhD	ICI + chemo	PD-1	NR/58	NR	0.46 (0.30–0.70)	NR
			chemotherapy		NR/63	NR		
POPLAR	2016	Louis Fehrenbacher	ICI	PD-L1	9/25	37.5	0.60 (0.31–1.16)	0.49 (0.22–1.07)
			chemotherapy		3/23	13		
RATIONALE 304 [30]	2021	Shun Lu, MD, PhD	ICI + chemo	PD-1	NR/74	NR	0.31 (0.17–0.57)	NR
			chemotherapy		NR/36	NR		
RATIONALE 307-1	2021	Jie Wang, MD, PhD	ICI + chemo	PD-1	33/42	78.6	0.50 (0.28–0.89)	NR
			chemotherapy		22/41	53.7		
RATIONALE 307-2	2021	Jie Wang, MD, PhD	ICI + chemo	PD-1	37/42	88.1	0.43 (0.23–0.78)	NR
			chemotherapy		22/41	53.7		

*ICI* immune checkpoint inhibitors, *ICI* *+* *chemo* ICI-combined chemotherapy.

### Network meta-analysis

#### Indirect comparison of ORR

**Fig. 1:**
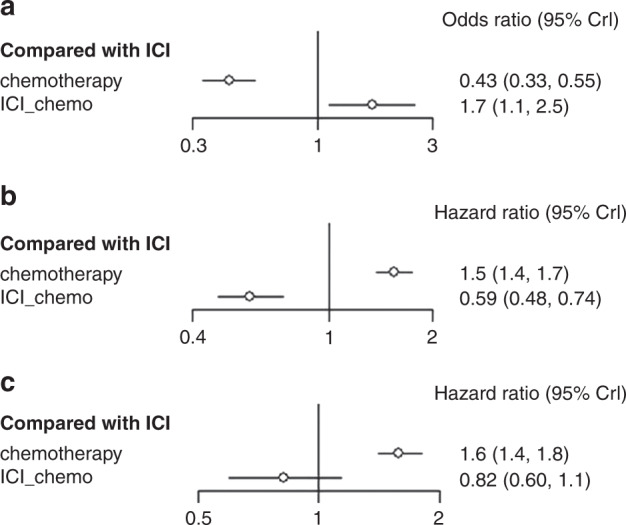
Forest plot of odds ratio or hazard ratios for ORR, PFS and OS in network meta-analysis. Forest plots presenting ORR (**a**) odds ratio analysis and PFS (**b**) and OS (**c**) hazard ratio analysis. ICI immune checkpoint inhibitors, chemo-ICI ICI-combined chemotherapy.

**Fig. 2:**
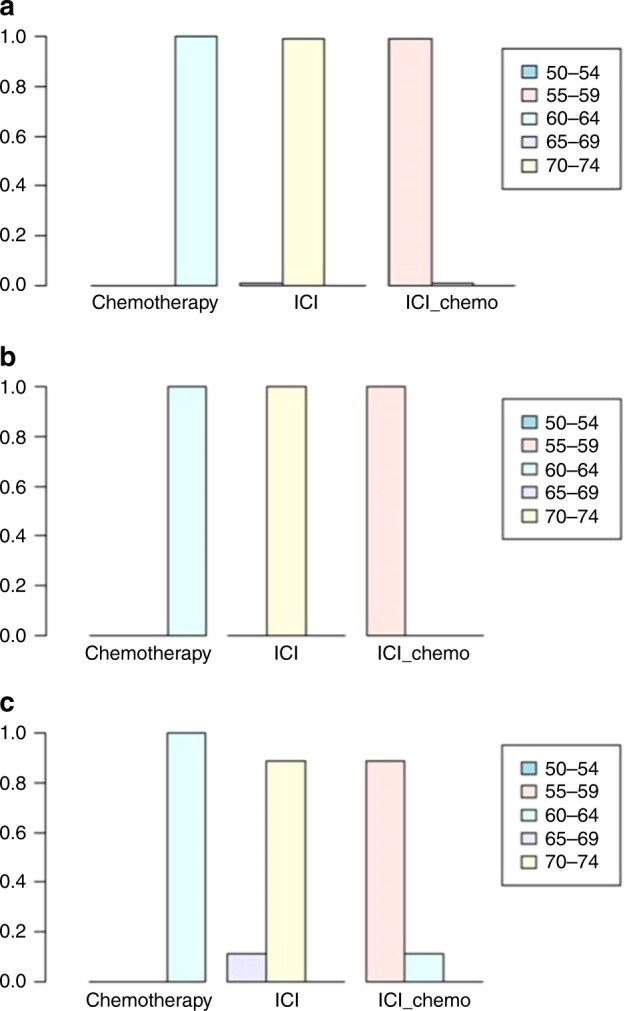
Ranking probabilities base on the multiple comparisons on ORR, PFS and OS in network meta-analysis. Ranking probabilities on ORR (**a**), PFS (**b**), and OS (**c**) in NSCLC patients with a high PD-L1 expression according to multiple comparisons. ICI immune checkpoint inhibitors, chemo-ICI ICI-combined chemotherapy.

The network plot displays the comparisons between each treatment (Supplemental Fig. 2). The OR was 2.3 (95% CI: 1.8–3.0) for ICI vs chemotherapy and 3.9 (95% CI: 2.8–5.5) for chemo-ICI vs chemotherapy. An indirect comparison between the two treatment protocols revealed that patients receiving chemo-ICI were more likely to respond than those receiving ICI (OR 1.7, 95 % CI 1.1–2.5) (Fig. 1a). When compared with chemotherapy, chemo-ICI exhibited a better effect than ICI (Table 2) in pairwise comparison. In Bayesian ranking, the curves indicated the probability of each treatment being ranked from first to last concerning OR and 95% CI. The chemo-ICI was the most possible therapy to be ranked first (Fig. 2a).

**Table 2:** Comparative efficacy of treatments for ORR in network meta-analysis.

Chemotherapy	2.32 (1.83, 3)	3.9 (2.78, 5.39)
0.43 (0.33, 0.55)	ICI	1.67 (1.11, 2.5)
0.26 (0.19, 0.36)	0.6 (0.4, 0.9)	ICI-chemo

*ICI* immune checkpoint inhibitors, *ICI-chemo* ICI-combined chemotherapy.

#### Indirect comparison of PFS

The HRs of PFS in patients with NSCLC who had high PD-L1 expression and were treated with ICI, chemo-ICI, or chemotherapy are shown in Fig. 1. Significant advantages in PFS (HR 0.59, 95% CI: 0.48–0.73) were observed for chemo-ICI over ICI. The Bayesian ranking results indicated the probability of chemo-ICI being ranked first (Fig. 2b). This result suggested that patients with NSCLC who had high PD-L1 expression may showcase more clinical benefit if they received chemo-ICI.

#### Indirect comparison of OS

The HRs of OS were 0.63 (95% CI: 0.55–0.71) for ICI vs chemotherapy and 0.52 (95% CI: 0.39–0.7) for chemo-ICI vs chemotherapy. Although a significant difference in OS was not observed for chemo-ICI when compared with ICI, an indirect comparison between the two treatment protocols signified that patients receiving chemo-ICI tended to have fewer progression events than those receiving ICI (HR 0.82, 95% CI 0.6–1.1, Fig. 1c). Furthermore, the result was confirmed by the ranking curves described on the basis of the Bayesian ranking results (Fig. 2c).

### Subgroup analysis

#### treatment line (first-line treated/previously treated)

**Fig. 3:**
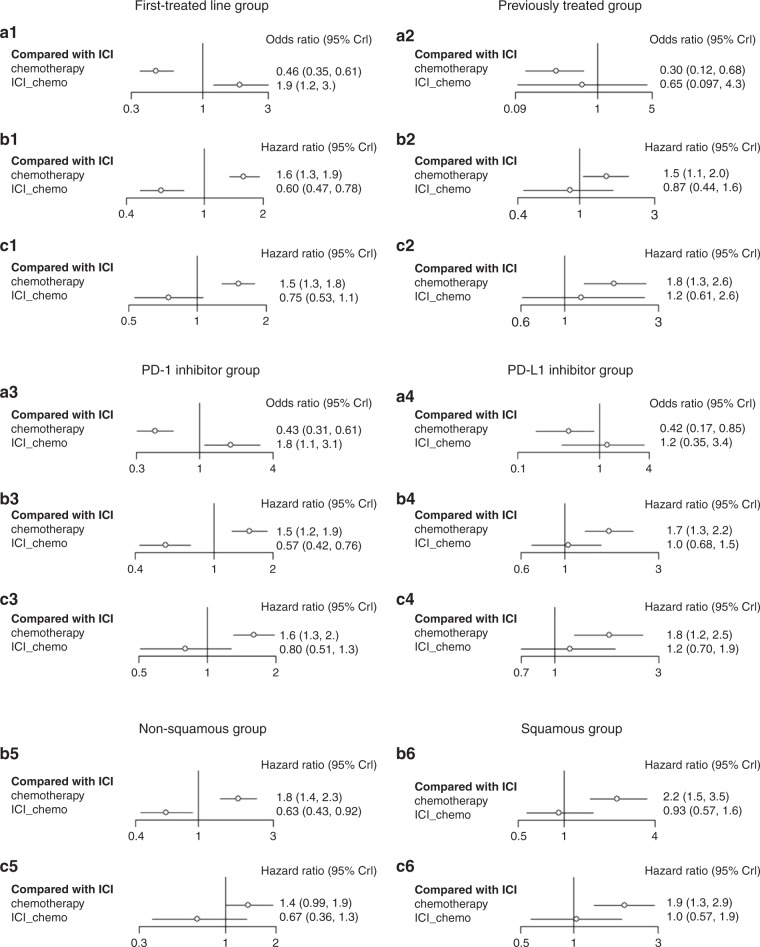
Forest plot of odds ratio or hazard ratios for ORR, PFS and OS in the subgroup analysis. Forest plots showing ORR (**a1**), PFS (**b1**) and OS (**c1**) hazard ratio analyses in the first-line treated group, ORR (**a2**), PFS (**b2**) and OS (**c2**) hazard ratio analyses in the previously treated group, ORR (**a3**), PFS (**b3**) and OS (**c3**) hazard ratio analyses in the PD-1 inhibitor group, ORR (**a4**), PFS (**b4**) and OS (**c4**) hazard ratio analyses in the PD-L1 inhibitor group, PFS (**b5**) and OS (**c5**) hazard ratio analyses in the non-squamous group and PFS (**b6**) and OS (**c6**) hazard ratio analyses in the squamous group. ICI immune checkpoint inhibitors, chemo-ICI ICI-combined chemotherapy.

**Fig. 4:**
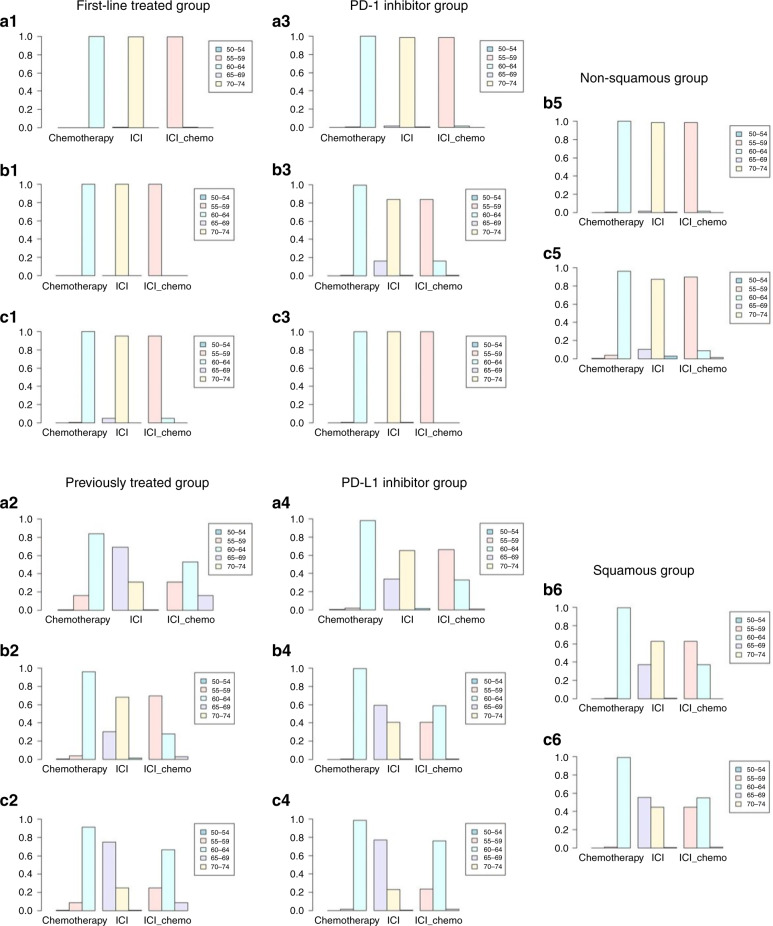
Ranking probabilities base on the multiple comparisons on ORR, PFS and OS in the subgroup analysis. Ranking probabilities based on the multiple comparisons on ORR (**a1**), PFS (**b1**) and OS (**c1**) in the first-line treated group, ORR (**a2**), PFS (**b2**) and OS (**c2**) in the previously treated group, ORR (**a3**), PFS (**b3**) and OS (**c3**) in the PD-1 inhibitor group, ORR (**a4**), PFS (**b4**) and OS (**c4**) in the PD-L1 inhibitor group, PFS (**b5**) and OS (**c5**) in the non-squamous group and PFS (**b6**) and OS (**c6**) in the squamous group. ICI immune checkpoint inhibitors, chemo-ICI ICI-combined chemotherapy.

In patients with NSCLC who had high PD-L1 expression and received PD-1 as first-line treatment, immense improvements in PFS (HR 0.60, 95% CI 0.47–0.78) and ORR (HR 1.9, 95% CI 1.2–3.0) were evident for chemo-ICI when compared with ICI (Fig. 3). Nonetheless, no significant difference was seen in OS (HR 0.75, 95% CI 0.53–1.1). The probability of each treatment ranking described according to the Bayesian ranking results showed that patients receiving ICI tended to endure more progression events than those receiving chemo-ICI (Fig. 4).

In patients with NSCLC who had high PD-L1 expression and were treated previously, PFS, OS and ORR were not different between chemo-ICI and ICI (Fig. 3). Based on the Bayesian ranking results, the probability of each treatment ranking further confirmed this result (Fig. 4).

#### PD-1 inhibitor/PD-L1 inhibitor

In patients with NSCLC who had high PD-L1 expression and were treated with a PD-1 inhibitor, significantly improved PFS (HR 0.63, 95% CI 0.43–0.92) and ORR (HR 1.8, 95% CI 1.1–3.1) were noticed in chemo-ICI when compared with IC (Fig. 3). However, no significant differences were perceived in OS (HR 0.8, 95% CI 0.51–1.3, Fig. 3). According to the Bayesian ranking results, the treatment ranking showed that patients receiving chemo-ICI may have better effects than those receiving ICI (Fig. 4).

In patients with NSCLC who had high PD-L1 expression and were treated with a PD-L1 inhibitor, PFS, OS and ORR were not different between chemo-ICI and ICI (Fig. 3). Furthermore, the results were presented based on the probability of each treatment ranking (Fig. 4).

#### Histological type (non-squamous/squamous)

In patients with non-squamous NSCLC who had high PD-L1 expression, chemo-IC was conspicuously superior to ICI in terms of PFS (HR 0.63, 95% CI 0.43–0.92). However, no significant difference in OS was noted (HR 0.67, 95% CI 0.36–1.3, Fig. 3). The probability of each treatment ranking described in accordance with the Bayesian ranking results demonstrated that chemo-ICI tended to be a better choice than ICI (Fig. 4).

In patients with squamous NSCLC who had high PD-L1 expression, both PFS and ORR were not different between chemo-ICI and ICI (Fig. 3). The treatment ranking in accordance with the Bayesian ranking results also confirmed this finding (Fig. 4).

## Discussion

The results of NMA revealed that chemo-ICI was associated with significantly improved ORR (OR 1.7, 95% CI 1.1–2.5) and PFS (HR 0.59, 95% CI: 0.48–0.73) when compared with ICI. Although significant differences in OS were not observed, an indirect comparison exposed that patients receiving ICI tend to experience more progression events than those receiving chemo-ICI (HR 0.82, 95% CI 0.6–1.1). In subgroup analysis, the non-squamous cohort, PD-1 inhibitor cohort, and first-line treatment cohort exhibited significant differences in ORR and PFS. In the case of OS, the indirect comparison demonstrated that patients receiving chemo-ICI tended to have a better effect than those receiving ICI. Nevertheless, in the squamous cohort, PD-L1 inhibitor cohort, and previously treated cohort, differences in PFS, OS, and ORR were not noted between chemo-ICI and ICI. These results provide instrumental evidence that a more individualised therapy is required in NSCLC.

In our squamous cohort subgroup analysis, we found no significant difference between PFS and OS, which may be attributed to the lack of inclusion of the related RCTs. Only subgroup analysis in impower lung-1 [14] and Keynote 042 [21] showed large examples and both involved studies comparing ICIs with chemotherapy. Hence, the examples of chemo-ICI vs chemotherapy were smaller than those of examples of ICI vs. chemotherapy. Moreover, only keynote 407 and impower 131 reported ORR dates, which were the date of chemo-ICI and chemotherapy, respectively, lacking ORR data of ICI and chemotherapy. The limited example and data may be the partial reason devoted to the results obtained.

In our PD-L1 inhibitor cohort subgroup analysis, we included 8 RCTs but only impower 131 and impower 132 [22] reported the data of chemo-ICI vs chemotherapy, with the examples being 91 and 45, respectively. Hence, the total example of ICI vs chemotherapy was larger than that of chemo-ICI vs chemotherapy. Meanwhile, both impower 131 and impower 132 RCTs compared atezolizumab plus chemotherapy with chemotherapy, while the others compared various ICIs to chemotherapy. These factors may have partially contributed to the unobserved differences in PFS, OS, and ORR.

In 2019, an indirect comparison meta-analysis evaluated the efficacy of pembrolizumab alone versus pembrolizumab combined chemotherapy for the first-line treatment of advanced NSCLC patients with PD-L1 TPS of ≥50%. The meta-analysis indicated that, for patients with NSCLC and high PD-L1 expression, the use of pembrolizumab combined chemotherapy as the first-line treatment may have had a better effect than that with pembrolizumab alone (ORR RRpem + chemo/pem 1.62, 95% CI 1.18–2.23 and PFS HRpem + chemo/pem 0.55, 95% CI 0.32–0.97) [23]. This result was verified by the current study. In contrast with the previously reported indirect comparison meta-analysis, we conducted a further subgroup analysis to explore the effect of histological type, treatment lines, and ICI type. Our meta-analysis included 22 RCTs, which, to our knowledge, is the most extensive NMA to date.

This study has several limitations. To start with, our meta-analysis depended on published results instead of individual patient data. Moreover, the antibodies used to test PD-L1 expression differed between the tests. The determination method of PD-L1 antibody detection used in RCT included in this paper is TPS and TC and IC calculation, respectively. The main difference between the different methods was based on whether they involved the calculation of the number of immunocytes with a positive expression in the tumour region. TPS means (number of pD-L1 membrane staining positive tumour cells/total tumour cells)*100%, TC means (PD-L1 membrane staining positive tumour cells at any strength/total tumour cells)*100% and IC means (PD-L1 membrane staining positive tumour-associated immune cells at any strength/total tumour-associated immune cells)*100%. Owing to the spatial and temporal heterogeneity in PD-L1 expression and the differences in detection mechanisms, PD-L1 was an imperfect biomarker [24]. However, PD-L1 expression in the tumour cells is currently the most widely applied biomarker for the stratification of patients [25]. Finally, data from head-to-head comparison of RCTs were lacking. We retrieved data about each treatment from subgroup analyses of RCTs. Some of the trials did not report OS and HRs and their 95% CIs for OS in their subgroup analyses. Besides, some trials had limited samples in the subgroup analysis of the high PD-L1 expression NSCLC cohort.

In summary, based on the results of our NMA, it could be stated that previously untreated patients with non-squamous NSCLC may show improved results if they accepted PD-1 treatment. Moreover, they may have longer progression-free survival and probably have more objective responses. In the future, the treatment of NSCLC is expected to get more precise, with a greater number of studies conducted on NSCLC patients under different situations. Our results provide evidence for the treatment of NSCLC patients with a high PD-L1 expression.

## Supplementary information

Reproducibility checklist

PRISMA NMA checklist

Supplemental Material

## Data Availability

All data generated or analysed during this study are included in this published article (and its supplementary information files).
