# Supplementary material for: Immune checkpoint inhibitors alone vs immune checkpoint inhibitors—combined chemotherapy for NSCLC patients with high PD-L1 expression: a network meta-analysis
Source: Br J Cancer. 2022 May 31;127(5):948–56. doi: 10.1038/s41416-022-01832-4 (PMC9427994; doi:10.1038/s41416-022-01832-4)
Supplement: Supplementary file 3 — Supplemental Material [file 41416_2022_1832_MOESM3_ESM.docx]

Supplemental Methods

Search strategies and number of studies yielded from each database.

PubMed：2847 Results

((non-small cell lung cancer[MeSH Terms]) OR (((non-small cell lung cancer[Title/Abstract]) OR (non-small cell lung carcinoma[Title/Abstract])) OR (NSCLC[Title/Abstract]))) AND((immune checkpoint inhibitor[Supplementary Concept]) OR(((((((((((((((immune checkpoint inhibitor) OR (PD-L1)) OR (programmed cell death-Ligand 1)) OR (PD-1 inhibitor)) OR (nivolumab)) OR (pembrolizumab)) OR (atezolizumab)) OR (avelumab)) OR (ticilimumab)) OR (durvalumab)) OR (Triprizumab)) OR (Cendilizumab)) OR (Carezumab)) OR (Tirelizumab)) OR (cemiplimab))) AND ((Randomized Controlled Trial[MeSH Terms]) OR(((clinical trial[Title/Abstract]) OR (RCT[Title/Abstract])) OR(study[Title/Abstract])))

Embase: 3063 Results

('non small cell lung cancer'/exp OR 'nsclc':ab,ti OR 'non-small cell lung cancer carcinoma':ab,ti) AND ('pd-l1'/exp OR 'programmed cell death-ligand 1':ab,ti OR 'pd-1 inhibitor':ab,ti OR 'immune checkpoint inhibitor':ab,ti OR 'nivolumab':ab,ti OR 'cemiplimab':ab,ti OR 'tirelizumab':ab,ti OR 'carezumab':ab,ti OR 'cendilizumab':ab,ti OR 'triprizumab':ab,ti OR 'durvalumab':ab,ti OR 'ticilimumab':ab,ti OR 'avelumab':ab,ti OR 'atezolizumab':ab,ti OR 'pembrolizumab':ab,ti) AND (Randomized Controlled Trial/exp OR clinical trial:ab,ti OR RCT:ab,ti)

Cochrane: 5549 Results

#1 MeSH descriptor: [Carcinoma, Non-Small-Cell Lung] explode all trees

#2 (NSCLC)

#3 (non-small cell lung cancer)

#4 (non-small cell lung carcinoma)

#5 #2 OR #3 OR #4

#6 (pd-l1)

#7 programmed cell death-ligand 1

#8 pd-1 inhibitor

#9 immune checkpoint inhibitor

#10 nivolumab

#11 cemiplimab

#12 tirelizumab

#13 carezumab

#14 cendilizumab

#15 triprizumab

#16 durvalumab

#17 ticilimumab

#18 avelumab

#19 atezolizumab

#20 pembrolizumab

#21 #6 OR #7 OR #8 OR #9 OR #10 OR #11 OR #12 OR #13 OR #14 OR #15 OR #16 OR #17 OR #18 OR #19 OR #20

#22 #1 OR #5 AND #21


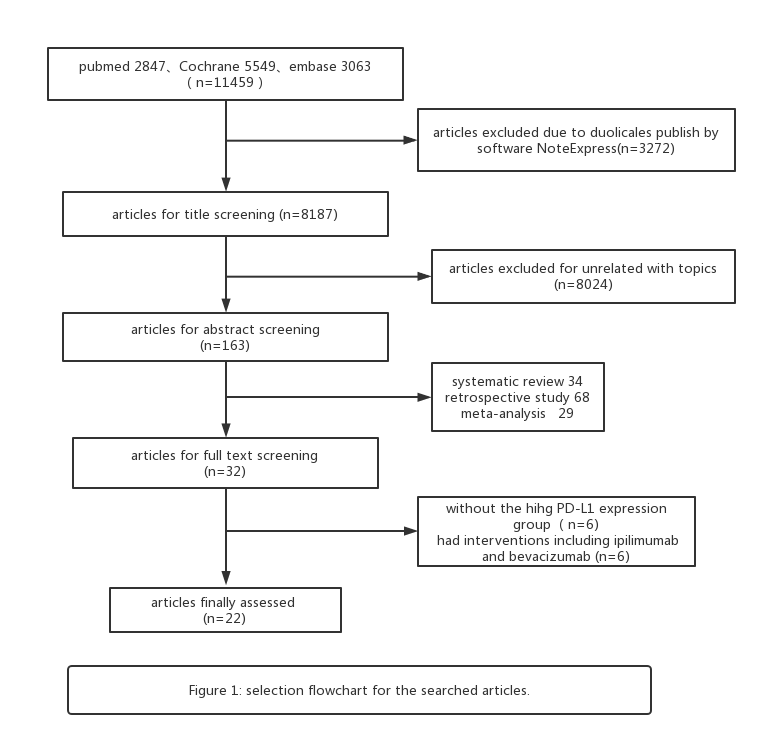


Supplemental figure 1：A selection flowchart for the searched articles.


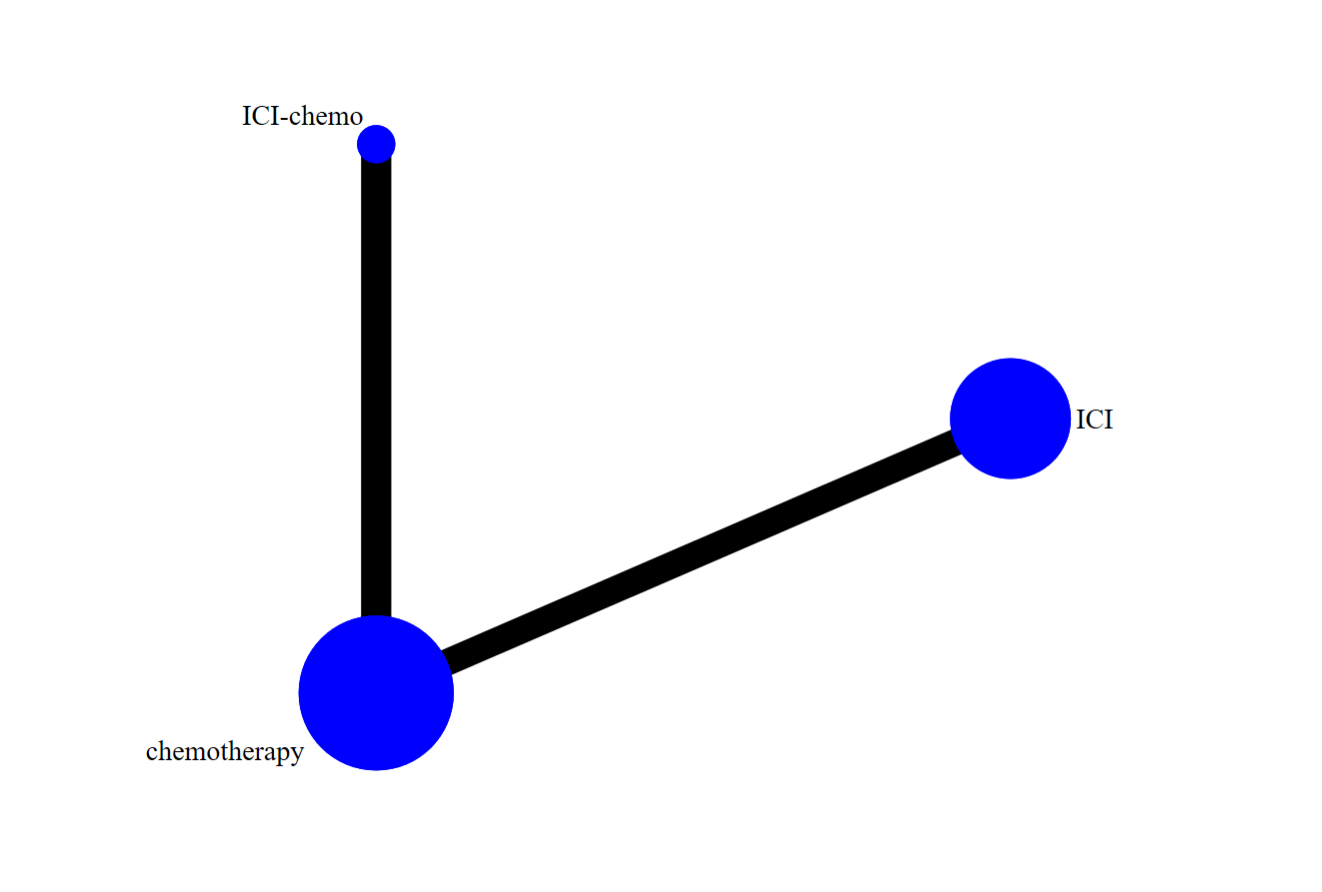


Supplemental figure 2: A network evidence plot for ORR. The size of the circle indicates the size of the sample, and the thickness of the line indicates the number of studies included. ICI: immune checkpoint inhibitors; chemo-ICI: ICI-combined chemotherapy.
